# Racism is not about “race”

**DOI:** 10.1038/s41598-023-47653-0

**Published:** 2023-12-15

**Authors:** Paola Bressan

**Affiliations:** Dipartimento di Psicologia Generale, University of Padova, 35131 Padua, Italy

**Keywords:** Human behaviour, Cultural evolution

## Abstract

Unfamiliar individuals are viewed with suspicion across the entire animal kingdom. This makes evolutionary sense, as outsiders may carry unfamiliar pathogens against which one has not yet developed immune defenses. In humans, the unfamiliar-pathogens idea has been dismissed on the grounds that people do not shun microbe-sharing contact with ethnic outgroups (other “races”) more than they do with ingroups. Reanalyzing the same public data on which such claims are based—6500 participants from China, India, USA, and UK—here I show that (1) people *do* behave as though the parasites of unfamiliar individuals were more dangerous, and (2) strangers’ ethnicity matters when, and only when, it is a proxy for unfamiliarity. This implies that racism could be tamed by acquainting our children with fellow humans of all shapes and colors, so that everyone in the world looks like family.

## Introduction

“*The foreigner living among you shall be to you as one born among you*”.

—*Leviticus 19:34.*

We animals tend to like individuals who belong to our own group (ingroup) better than those who do not (outgroup). Bees do it^1^, rats do it^2^, and however utterly wrong this seems to many of us, we humans do it too^3,4^. We value members of our own family, kin, village, country, ethnicity, “race”, and species more highly than members of other ones; we practice, alas, all manner of nepotism, tribalism, nationalism, ethnocentrism, racism, and speciescentrism.

Being so firmly rooted and widespread among creatures, this preference for one’s community members is exceedingly unlikely to have arisen by accident. A sensible, biologically sound explanation is that we have evolved to prefer contact with the ingroup, over the outgroup, because we are better placed to combat parasites that are ubiquitous in our own community as opposed to far-away ones (Ref.^5–7^). First, we have coevolved with such parasites for longer and may thus be more finely adapted to them (Ref.^5,7,8^; but see Ref.^9–11^). Second, after infecting all the susceptible individuals in our group, some viruses might no longer be around but be reintroduced by contact with foreigners^6,12^. Third, our immune cells are known to respond best to pathogens they have encountered before (Ref.^13^; see also^9,14^). The novel parasites carried by individuals coming from other communities, then, are bound to prove more dangerous to us than the old, familiar ones hosted by our neighbors.

**Figure 1:**
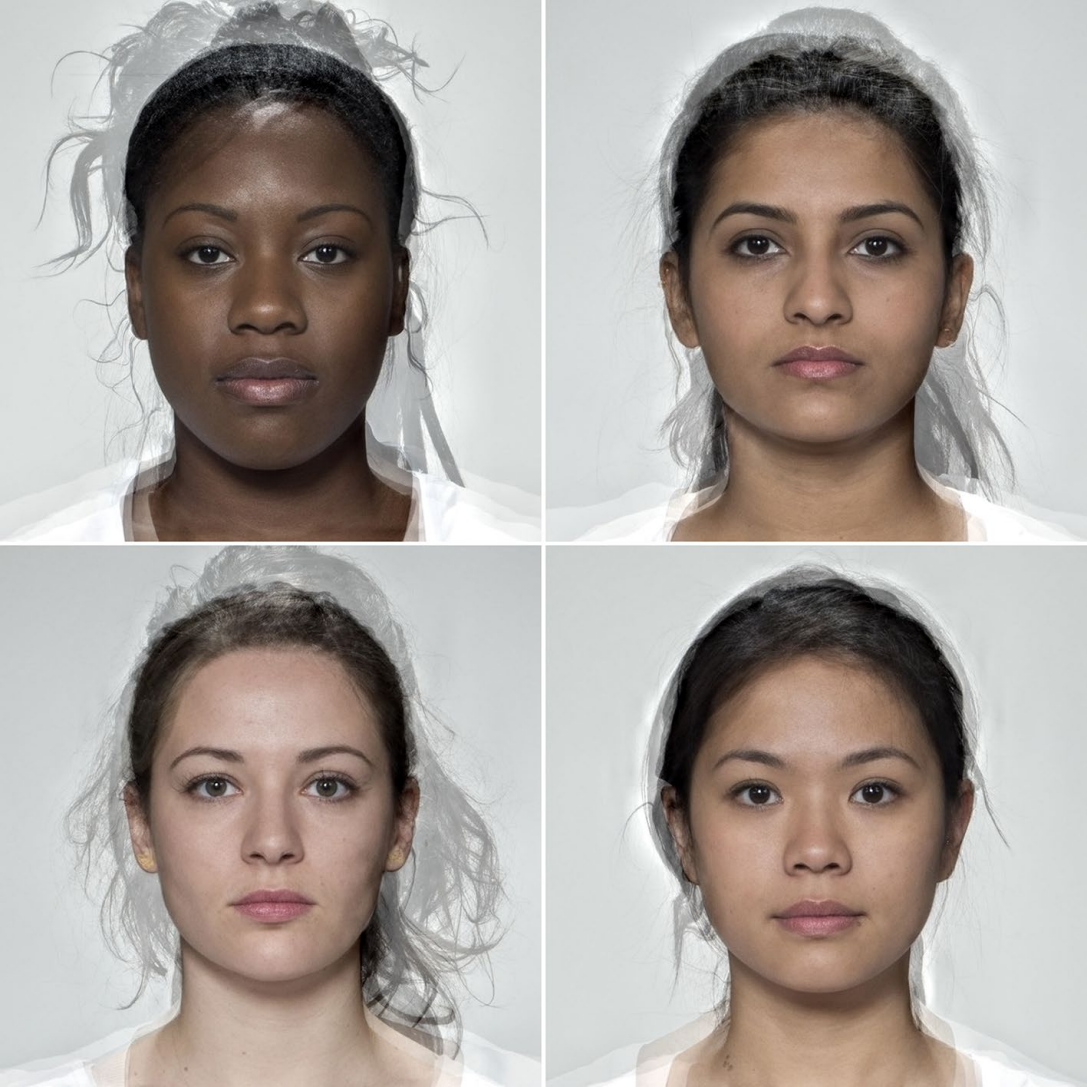
As our faces have adapted to different places and climates, we have evolved to interpret the degree of unfamiliarity in others’ features as a rough cue of geographical distance. Composite images (4 individuals per composite) of women of African, West Asian, White, and East Asian ethnic backgrounds. Original photos were taken in London. CC BY 4.0^17^.

Although it never raises any objections when applied to other animals, for some reason this argument has not gone down well in the case of humans. Two recent, large works (Ref.^15^^,^^16^) have indeed claimed to provide direct evidence against the notion that “the mind has evolved to interpret outgroup membership as a cue to pathogen threat”^16^. This conclusion hinges on the finding that people do not appear to dislike close (“microbe-sharing”) contact with ethnic outgroups more than they do with ethnic ingroups. The assumption being, of course, that individuals of ethnicities other than one’s own are the most natural sort of human outgroups (Fig. 1).

Here, I show that the very same data that are purported to speak against the unfamiliar-pathogens idea actually support it. I make my point by reanalyzing the publicly available data of both studies. Participants rated how comfortable they felt at being in close contact with a stranger whose face, portrayed in a photo, did or did not display a severe infection. The stranger did or did not share the participant’s ethnicity (European-ancestry vs. dark-skinned Indian-ancestry faces in the first study, conducted in the USA and India^15^; European-ancestry vs. Chinese-ancestry faces in the second, conducted in the UK and China^16^).

A previous reanalysis^18^ I carried out of the US/India Study’s data has shown that, regardless of their ethnicity, strangers who look less similar to one’s community members are perceived as sicker, and people are less willing to touch them. In the current work I look instead at the specific role of ethnicity, drawing on the data from all four countries: United States of America, United Kingdom, India, and China. It so happens that the first three are among the most multiethnic in the world—hosting as they do people of vastly different ancestries, with skin colors ranging from white to black—whereas China is one of the most ethnically homogeneous, as 92% of its population belongs to the Han ethnic group and virtually all minorities are still of East Asian descent. I show, for the first time, that people of another ethnicity trigger xenophobia in monoethnic communities (where they are rarely ever met) and xenophilia in multiethnic ones (where they are encountered all the time); and that conspicuous signs of infection increase the xenophobia and decrease the xenophilia.

Along with the earlier ones, the findings presented here paint a full, within- and between-societies picture of stranger avoidance in our species. They leave little doubt that, just as predicted by the unfamiliar-pathogens theory, (1) like other animals, we use unfamiliarity as a cue to infectiousness, and (2) ethnic outgroupness matters only to the extent that it reflects unfamiliarity; otherwise, it does not matter at all.

## Methods

Here I reanalyze publicly available data from 6488 participants^15,16^ recruited via the online platforms Amazon Mechanical Turk (India and USA), WJX (China), and Prolific (UK).

In the US/India Study^15^, participants looked at a photograph showing the face of a man, who could be White or Indian (dark-skinned). The face either carried an infection cue (a facial rash added digitally to the image) or appeared “healthy” (that is, had no rash added to it). In the UK/China Study^16^, participants saw a photograph showing the face of a man or a woman who could be either White, and described as a Briton living in the UK, or East Asian, and described as a Chinese living in China. The face either displayed an infection cue looking like shingles, or appeared “healthy” (unmodified), or wore a surgical mask. In both studies, participants were asked a few questions about the person in the photo and indicated how comfortable they would feel about interacting closely with him or her.

**Figure 2:**
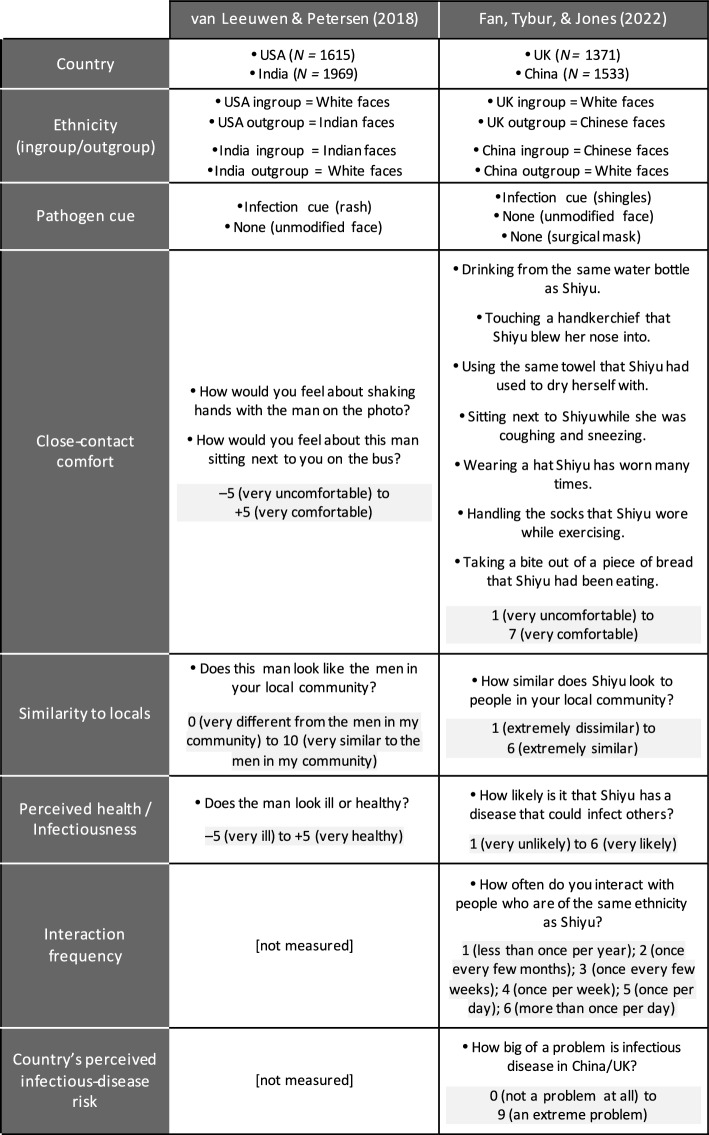
Variables analyzed in this article and the way they were measured in the US/India and UK/China studies. In the latter, the person in the photo was labeled with a name typical of his or her purported country of origin (here, Shiyu).

**Figure 3:**
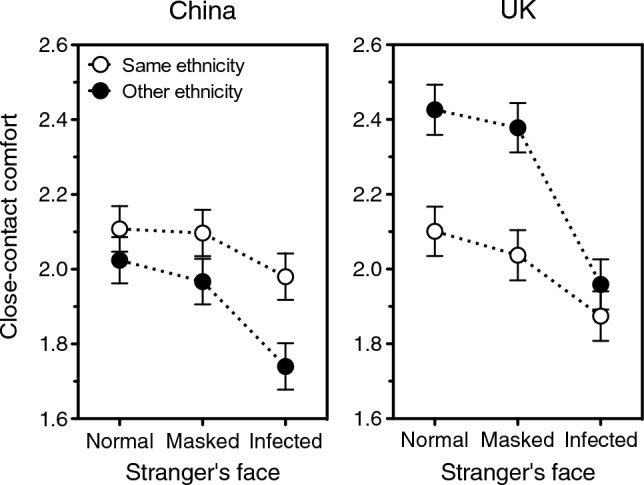
Close-contact comfort with a stranger who either does (open symbols) or does not (solid symbols) share one’s ethnicity, in China (left panel) and UK (right panel). Comfort is plotted as a function of whether the stranger appears healthy, wears a face mask, or shows a severe infection cue. Error bars indicate one standard error of the mean. Data source: UK/China Study^16^.

**Figure 4:**
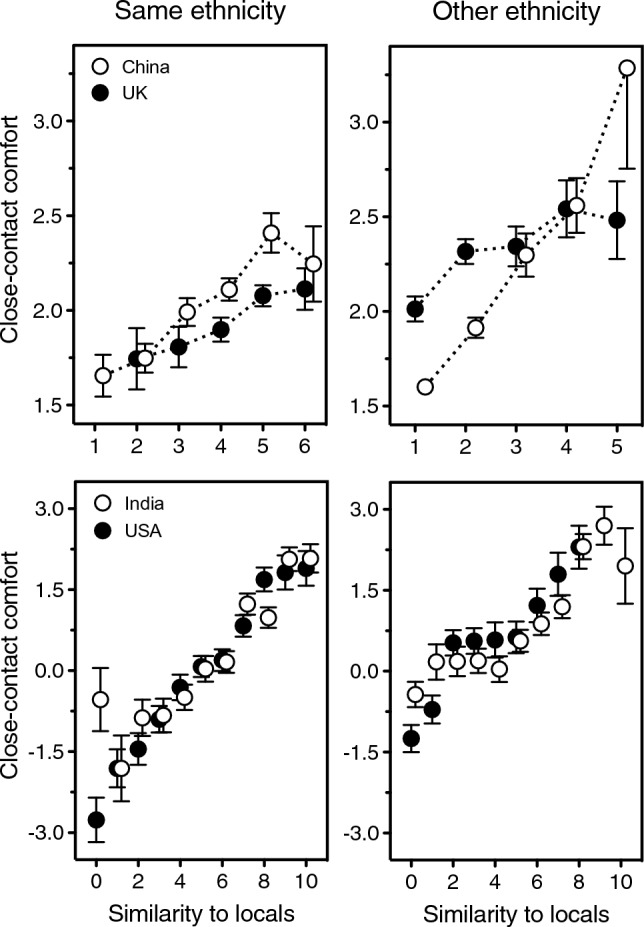
Close-contact comfort with a stranger who either does (left panels) or does not (right panels) share one’s ethnicity, as a function of how similar he or she looks to the people in one’s local community. Error bars indicate one standard error of the mean. Top: Participants from China (open symbols) and from the UK (solid symbols) in the UK/China Study. Bottom: Participants from India (open symbols) and from the USA (solid symbols) in the US/India Study. Data points relying on only 10 or fewer ratings are not represented (these are the “1 = extremely dissimilar from locals” endpoint for same-ethnicity faces in UK and the “6 = extremely similar to locals” endpoints for other-ethnicity faces in both China and UK). Open symbols have been nudged horizontally to diminish overlap and ensure visibility of all data points.

**Figure 5:**
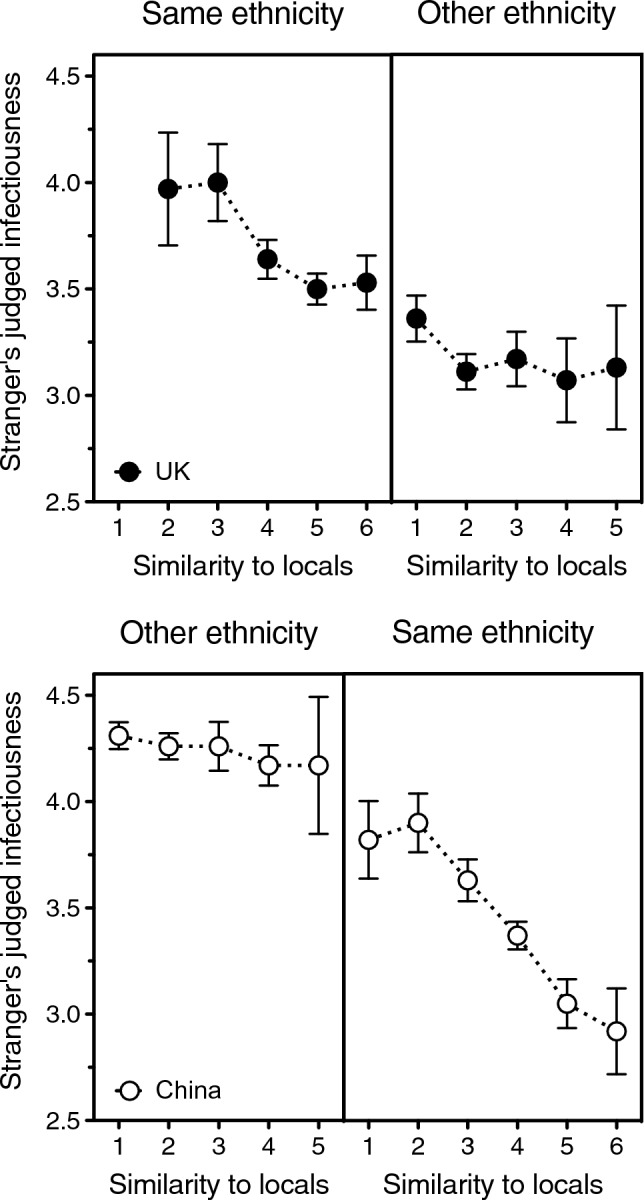
Judged likelihood that a stranger has an infectious disease as a function of how similar he or she looks to the people in one’s local community, in the UK (top, solid symbols) and China (bottom, open symbols). Data are plotted separately for a stranger who either does (top, left panel; bottom, right panel) or does not (top, right panel; bottom, left panel) share one’s ethnicity. Note that, in the top and bottom graphs, the left and right panels have been transposed relative to one another so as to highlight the reversed symmetry between the curves. Error bars indicate one standard error of the mean. Data points relying on only 10 or fewer ratings are not represented (these are the “6 = extremely similar to locals” endpoints for other-ethnicity faces in both countries, and the “1 = extremely dissimilar from locals” endpoint for UK same-ethnicity faces). Data source: UK/China Study^16^.

**Figure 6:**
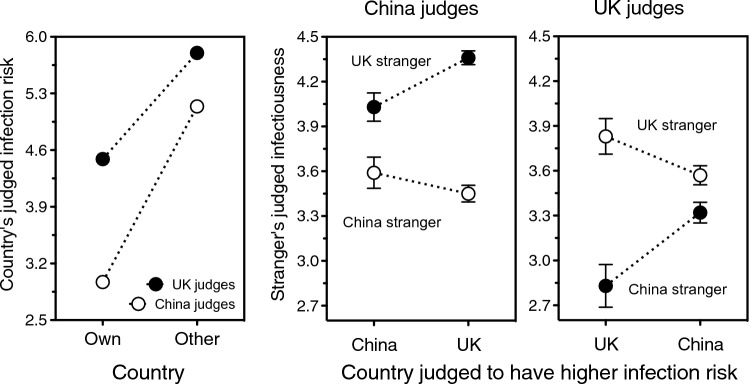
Perceived infectiousness of strangers as a function of whether their country of origin is believed to have a higher or lower infection risk than one own’s. Left panel: Risk of infectious disease in the United Kingdom and in China, as judged by British (solid symbols) and Chinese (open symbols) participants. Error bars, indicating one standard error of the mean, are smaller than the symbols. Middle and right panels: Likelihood, as judged in China (middle panel) and UK (right panel), that a stranger who either does (open symbols) or does not (solid symbols) share one’s ethnicity carries an infectious disease. Data are plotted as a function of which country, one’s own or the other, is deemed to have a higher infection risk. Error bars indicate one standard error of the mean. Data source: UK/China Study^16^.

**Figure 7:**
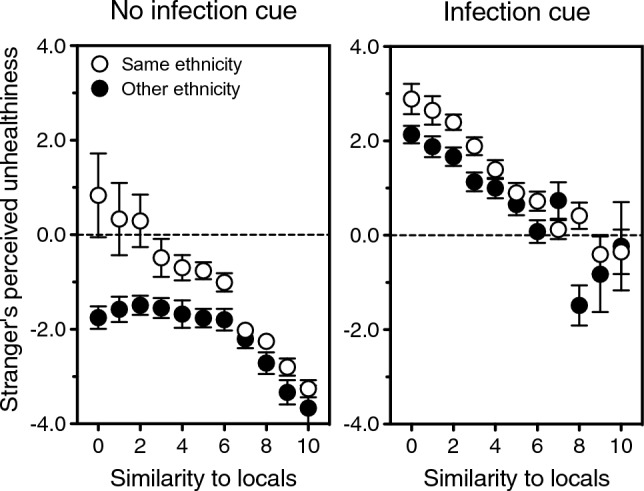
Perceived unhealthiness of a stranger who either does (open symbols) or does not (solid symbols) share one’s ethnicity, as a function of how similar he or she looks to the people in one’s local community. Error bars indicate one standard error of the mean. Left panel: Stranger with no visible infection cue. Right panel: Stranger with a visible infection cue. Data source: US/India Study^15^.

**Figure 8:**
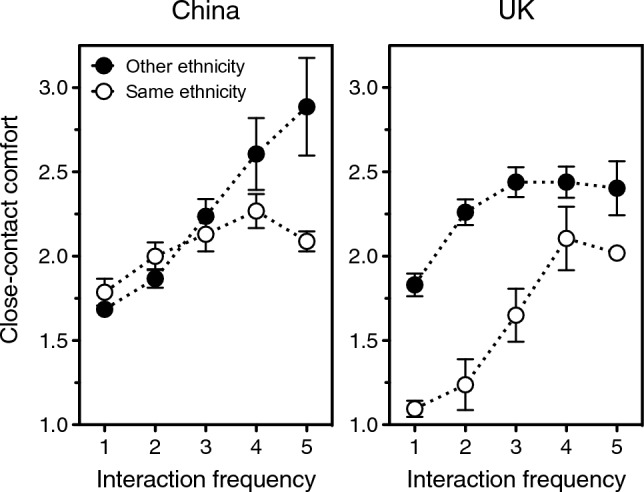
Close-contact comfort with a stranger who either does (open symbols) or does not (solid symbols) share one’s ethnicity, in China (left panel) and UK (right panel). Comfort is plotted as a function of one’s frequency of interaction with people of the stranger’s ethnicity, from 1 = less than once per year to 5 = every day. (The latter category collapses the two original categories “Once per day” and “More than once per day”; even so, in the other-ethnicity condition this merged category includes only 6% of responses overall.) Error bars indicate one standard error of the mean. Data source: UK/China Study^16^.

All variables of interest are presented in Fig. 2, along with the exact way each was measured in the two studies. None of the data were excluded or transformed for the analyses presented here. The authors of the original articles performed on their own data entirely appropriate analyses; nonetheless, they do not appear to have carried out—in the same form as here, or at all—any of the analyses whose results are depicted in this paper (Figs. 3, 4, 5, 7, 8, and middle and right panels of Fig. 6). In particular, in the US/India Study, Similarity to locals and Perceived health were introduced as manipulation checks and not used in the analyses themselves.

## Results

The unfamiliar-pathogens theory posits that what drives our avoidance of strangers is our unfamiliarity with their parasites^18^. Only if strangers’ ethnicity is a proxy for their unfamiliarity should our “behavioral immune system”^19^ care at all about it. Hence, the theory predicts that “true” outgroupness (that is, perceived unfamiliarity^18^) will decrease our willingness to share strangers’ parasites (that is, comfort with close contact) everywhere, but a mere ethnicity difference will not. Members of other ethnicities automatically qualify as outgroup only in ethnically homogeneous communities. So, it is in such communities that they should instinctively be seen as health threats.

The data examined here were collected in four countries: the multiethnic United States of America, United Kingdom, and India, and the monoethnic China. In multiethnic societies, where people are continually exposed to both individuals who share their own ethnicity and others who do not, the pathogens of ethnic ingroups and outgroups are equally likely to have been encountered before. The costs of contact with either group are thus very similar. Benefits are hardly the same, however, as interaction with outsiders favors (or, more correctly, did so during the bulk of our evolutionary history) the acquisition of new ideas, technologies, and innovations—expanding, at the same time, one’s networks for trading, marriage, and all manner of social alliances^5,7^. And yet, if one’s willingness to risk catching other people’s pathogens reflects this benefit–cost ratio, one’s preference for interesting foreigners over ordinary neighbors should drop precipitously as soon as such pathogens are no longer hypothetical but advertise themselves in full colors.

These predictions were tested by running, on the close-contact comfort ratings of the UK/China Study^16^, an ANOVA with Pathogen cue (none, visible infection), Ethnicity (ingroup, outgroup), and Country (China, UK) as factors. (Conservatively, the face-mask data were placed in the “no visible infection” condition. Indeed, these data were collected during the COVID-19 pandemic, in January 2022, when in both China and the UK face masks were mandatory in many public settings and hence provided no reliable signal, whether direct *or* indirect, that the wearer could be carrying an infection.)

First, people preferred contact with ethnic *outgroups* in multiethnic UK and ethnic *ingroups* in monoethnic China, Ethnicity × Country: *F*(1, 2896) = 23.78, *p* < 0.0001. Second, a visible infection caused a steeper decrease in contact comfort when it was carried by ethnic *outgroups* as opposed to ingroups, Ethnicity × Pathogen cue: *F*(1, 2896) = 5.88, *p* = 0.015, and this was the case in both societies, Ethnicity × Pathogen cue × Country: *F* < 0. That is, signs of infection increased xenophobia in China and decreased xenophilia in the UK. Figure 3 unpacks this general effect by depicting how comfortable people felt at close range with a person of the same (open symbols) or another (closed symbols) ethnicity who either appeared healthy, wore a face mask, or displayed an active infection. In the left panel (China), open symbols always sit higher than solid ones, but the gap between the rightmost ones is by far the largest; in the right panel (UK), open symbols always sit lower than solid ones, but the gap between the rightmost ones is by far the smallest.

The same ANOVA was carried out on the close-contact comfort ratings of the US/India Study^15^. Like in multiethnic UK, xenophilia dropped in the presence of a visible infection, Ethnicity × Pathogen cue: *F*(1, 3648) = 5.09, *p* = 0.024, in both multiethnic India and multiethnic USA, Ethnicity × Pathogen cue × Country: *F* = 1, *p* = 0.303.

Thus, the data fully support the unfamiliar-pathogens theory’s prediction that ethnic outgroupness will drive contact comfort in opposite directions in monoethnic and multiethnic societies. They also corroborate the theory’s prediction that signs of infection should reduce comfort more sharply when they crop up on a human of another ethnicity, as opposed to one’s own. Note that this reduction in comfort is expected to occur in both types of societies, but for different reasons. In a multiethnic community, where one comes across both same- and other-ethnicity people all the time, everybody’s pathogens are equally nasty. A visible infection simply levels the playing field, abating any extra advantages of interacting with one group over the other (Fig. 3, right panel). In a monoethnic society, however, a different ethnicity is a resonant cue of unfamiliarity. Interaction with foreigners still has its perks, but when one does business with someone who is carrying an obvious disease, benefits stay the same and risks soar. Crucially, this surge is greater if the infection is new and unknown rather than old and tested; that is, if the stranger does not share the community members’ ethnicity (Fig. 3, left panel).

And yet, it is a crucial prediction of the unfamiliar-pathogens theory that others’ ethnicity should matter only insofar as it reflects one’s lack of familiarity with their parasites (their true degree of “outgroupness”^18^). In humans and other animals, a good indication of the familiarity of unknown others is how much they resemble the conspecifics in one’s local community^18,20,21^. The importance of this resemblance comes up very clearly in both the US/India and UK/China studies: one’s comfort at touching strangers—or sharing their personal stuff—rises with their similarity to locals (Fig. 4). Although similarity to locals was measured on two different scales (11 points in USA and India as opposed to only 6 in China and UK), and the US/India Study also had a larger sample (3619 vs 2904 participants), the effect replicated in all four countries, within both ethnic ingroups and outgroups (USA, ingroup: beta = 0.49, *p* < 0.0001; USA, outgroup: beta = 0.31, *p* < 0.0001; India, ingroup: beta = 0.37, *p* < 0.0001; India, outgroup: beta = 0.30, *p* < 0.0001; China, ingroup: beta = 0.20, *p* < 0.0001; China, outgroup: beta = 0.37, *p* < 0.0001; UK, ingroup: beta = 0.10, *p* = 0.009; UK, outgroup: beta = 0.14, *p* = 0.0002).

If the pathogens of unfamiliar individuals are more dangerous to us than those of familiar ones, our built-in assumption that strangers are infectious should be stronger for outgroup than for ingroup members^18^. Indeed, being more afraid of disease is linked to stronger negative feelings toward foreigners but not compatriots (shown in Poland^22^) and against unfamiliar but not familiar immigrants (shown in Canada^23^; see also^24–29^). This implies that people of another ethnicity should be perceived as more infectious than people of one’s own in China, but not in India, UK, or USA. Each study adopted its own infectiousness measure: either the stranger’s apparent health (US/India Study) or the stranger’s likelihood of carrying an infectious disease (UK/China Study). In the US/India Study, just as expected from an unfamiliar-pathogens perspective, Ethnicity had no significant effects on perceived health, either on its own (*F* < 1) or in interaction with Country (*F* = 1.2, *p* = 0.26). Yet in the UK/China Study, again as expected, Ethnicity was instead significant and interacted robustly with Country, *F*(1, 2900) = 176.49, *p* < 0.0001. That is, being of another ethnicity increased a stranger’s perceived infectiousness in monoethnic China, *r* = 0.34, *p* < *0.0*001, and decreased it in multiethnic UK, *r* = – 0.15, *p* < *0.0*001.

This large, impressive reversed symmetry is depicted in Fig. 5. In the UK (top), Chinese strangers (“other ethnicity”, on the right) looked even less infectious than did the most familiar-looking Britons. In China (bottom), in striking contrast, UK strangers (“other ethnicity”, on the left) looked more infectious than did the most unfamiliar-looking among the Chinese. To put it crudely, in a monoethnic society, members of another “race” are perceived as extreme pathogen threats: super-outgroups. And yet, *provided* one is regularly exposed to their parasites, people of other “races” are clearly seen as worthier to get close to than are people of one’s own. Indeed, the data suggest that the infection risk that is inevitable in every human interaction is downplayed in front of strangers who are better placed to expand one’s relationship networks, bringing in the benefits of social and cultural diversity (Fig. 5, top).

Incidentally, the perceived benefits of contact with different ethnicities also turn up in these datasets. In the USA/India Study, White and Indian faces appeared no different in any other traits, but Indians rated Whites as wealthier than fellow Indians, *r*(2002) = 0.15, *p* < 0.0001, and Americans rated Indians as more competent (intelligent and capable) than fellow Whites, *r*(1620) = 0.15, *p* < 0.0001. In the UK/China Study, the only available data concern perceived generosity (“prosociality”), and indeed, Britons rated Chinese as more generous than fellow Britons, *r*(1371) = 0.10, *p* = 0.0003. The only exception to this general endorsement of outgroup assets was in fact the Chinese rating of Britons as marginally *less* generous than fellow Chinese, *r*(1533) = – 0.06, *p* = 0.021.

Tellingly, the underestimation of ethnic strangers’ infectiousness found in multiethnic UK occurs even when the risk of infectious disease appears larger in the stranger’s place of origin than in one’s own nation. The latter is the case in both countries: Chinese participants find the UK more dangerous than China, paired-samples *t*(1532) = 27.4, *p* < 0.0001, and UK participants find China more dangerous than the UK, *t*(1370) = 20.5, *p* < 0.0001 (Fig. 6, left panel). Note that—consistent with the idea that the deployment of evolved behaviors should flexibly incorporate current, relevant information—the estimated infection risk in the other country relative to one’s own *does* sway one’s attitude towards other-ethnicity strangers. It raises their apparent infectiousness, *r* = 0.18, *p* < 0.0001, and reduces close-contact comfort with them, *r* = – 0.16, *p* < 0.0001. In monoethnic China, however, UK faces look more infectious than Chinese ones even to people who see the UK as a safer place (Fig. 6, middle panel: solid symbols are always higher than open ones). In multiethnic UK, conversely, Chinese faces look less infectious than UK ones even to those who judge China to have a larger infection risk than the UK (Fig. 6, right panel: solid symbols are always lower than open ones).

The xenophilia found in the British sample is hardly a fluke, as it comes up in the American and Indian ones too: at least to the particular participants in these studies, people of the other ethnicity looked healthier (Fig. 7). When they bore no visible pathogen cue, they looked in fact much healthier than unfamiliar individuals of one’s own ethnicity. (Fig. 7, left panel: solid symbols sit lower than open ones, but the gap becomes progressively smaller moving to the right, that is, as familiarity increases. Ethnicity: beta = – 0.57; Similarity to locals: beta = – 0.56; Ethnicity × Similarity to locals: beta = 0.38, all *p*_s_ < 0.0001.) Only in the presence of an active infection was the effect of ethnicity markedly reduced, and even then not entirely eliminated. (Fig. 7, right panel: solid symbols sit slightly lower than open ones. Ethnicity: beta = – 0.14, *p* = 0.001; Similarity to locals: beta = – 0.40, *p* < 0.0001; Ethnicity × Similarity to locals: beta = 0.04, *p* = 0.344.) This pattern—people’s xenophilic reactions to ethnically diverse society members being dampened by a visible pathogen cue—is very similar to that found for close-contact comfort in the UK (right panel of Fig. 3): there, too, social diversity lost its perks in front of clear signs of infection.

It goes without saying that the openness to new experiences that drives xenophilia may well be above average in individuals who, like these studies’ participants, habitually engage in online surveys via professional crowdsourcing platforms. If so, note that the general population may display weaker xenophilia in multiethnic communities; and yet, by the same token, also stronger xenophobia in monoethnic ones.

Finally, a parasite-familiarity cue that is somehow complementary to a stranger’s resemblance to locals is one’s own frequency of contact with people who resemble the stranger. From an unfamiliar-pathogens stance, this variable is bound to reflect one’s degree of exposure to pathogens typical of the stranger’s group. In the UK/China Study, participants were asked how often they “interacted” with people of the same ethnicity as the stranger (Fig. 2). Unfortunately, this formulation is far from ideal: people can infect one another even when they do not interact, but merely touch the same surfaces or inhale the air others have just exhaled (e.g. Ref.^30^). Therefore, this variable is a rather poor proxy for parasite-sharing contact with people of one’s own ethnicity, who are typically always around. In fact, it is also a feeble proxy for contact with people of other ethnicities in multiethnic societies such as the UK—where most individuals stand next to members of other ethnicities virtually every day (in public places, on public transport, in shops, at school), whether or not they deliberately “interact” with them.

In fact, inspection of responses shows that this question is unlikely to have been interpreted as the authors intended. In particular and quite strikingly, whereas about 90% of UK participants reported interacting with people of the same ethnicity as the European-ancestry face every day (usually “more than once per day”), nearly 50% of Chinese participants reported interacting with people of their purported ethnic ingroup only “once every few weeks” *or less*. I can see two possible explanations for this remarkable oddity in the data. First, in the Chinese questionnaire the verb “to interact” is translated with the word 接触, which as a noun means “touching” or “contact”, and as a verb “to contact” or “to be in touch”. Because this Chinese word evokes the idea of *physical* contact far more than does the English “interact” (P. Kramer, personal communication, 22 September 2022), contact frequency may have been interpreted differently in the two societies. Second, as China is a huge country, featuring an extreme contrast in climate between its northern and southern regions, some geographically separated populations have evolved slightly different traits. The typical northern and southern Chinese, for example, bear distinctive facial characteristics (see also Ref.^31^). In China, thus, some of the Chinese faces meant to represent the ethnic ingroup could have easily been regarded by many as ethnic *outgroup* members instead.

Still, although it would be best not to make much of the fine details, one’s frequency of interaction with people of the same ethnicity as the stranger does tend to increase one’s readiness to touch his or her dirty handkerchief, drink from the same bottle, and so on (China, ingroup: *r*_S_ = 0.10, *p* = 0.006; China, outgroup: *r*_S_ = 0.27, *p* < 0.0001; UK, ingroup: *r*_S_ = 0.09, *p* = 0.018; UK, outgroup: *r*_S_ = 0.20, *p* < 0.0001). Means are depicted in Fig. 8. And, in monoethnic China but not in multiethnic UK, one’s frequency of interaction with people of the same ethnicity as the stranger decreases his or her perceived infectiousness (China, ingroup: *r*_S_ = – 0.38, *p* < 0.0001; China, outgroup: *r*_S_ = – 0.11, *p* = 0.002; UK, ingroup: *r*_S_ = 0.005, *p* = 0.69; UK, outgroup: *r*_S_ = – 0.07, *p* = 0.68).

These results are consistent with social psychologists’ robust finding that intergroup contact lessens prejudice (see Ref.^32^ for a large meta-analysis). Indeed, infants as young as 3 months of age already show a visual preference for what the authors call “own-race” faces relative to “other-race” ones, but only if they live in a mostly homogeneous “own-race” environment^33^: exposure to “other-race” faces can erase this bias^34^ or overturn it^35^. Such effects have been attributed to the “tendency for familiarity to breed liking”^32^. It has been suggested that familiarity breeds liking because familiar people are liked better than unfamiliar ones, and such feelings “overgeneralize” to strangers who resemble known individuals^20,21^. Yet this umbrella explanation is at odds with the fact that, in several notable departments (such as mate choice, e.g.^36, 37^) and in humans just as much as in other animals, it is *un*familiarity that breeds liking. But most importantly, the overgeneralization account begs the question of why we animals prefer familiar individuals in the first place. The unfamiliar-pathogens theory provides one strong, simple, biologically grounded reason for why, in the specific context of relationships between groups, we do.

## Discussion

Just like do other animals, then, people perceive parasites as a larger threat when they are carried by individuals who look less familiar: this is what the deepest root of “outgroupness” may be all about^18^. Outgroup avoidance is, alas, only natural, as pathogens to which one has not been previously exposed truly are more dangerous^13^. These cross-cultural data^15,16^ show that strangers’ unfamiliarity (their dissimilarity from our community members) make us less willing to share their parasites: to shake their hand, sit next to them on the bus, sit next to them while they are coughing and sneezing, touch their dirty handkerchiefs, towels, socks, and hats, drink from the same bottle and bite into the same bread. And yet, these data also show that our fellow humans’ ethnicity, by itself, has nothing to do with ingroupness and outgroupness: foreigners (*and* compatriots!) are seen as outgroup members if, and only if, they look unfamiliar. Rather than failing to support the unfamiliar-pathogens idea (as claimed by the authors of the original studies^15,16^), the basic irrelevance of ethnic outgroupness actually provides clear, strong, independent evidence for it. Indeed, of all alternative theories of outgroup avoidance advanced so far, the unfamiliar-pathogens one is the only one consistently backed up by the data (for broad reviews of the evidence, see Ref.^3,5,7^; for a direct comparison between competing theories, see Ref.^18^).

Converging evidence from two independent studies and four datasets, comprising alternative and complementary measures of outgroupness and infectiousness, speak clearly for the idea that we use outgroupness as a cue to infectiousness. And yet, such a strategy can successfully evolve only if ingroupness and outgroupness are not inherent properties of others—but superficial, temporary, adjustable tags, based on the looks of individuals to whose parasites we are, or are not, habitually exposed. Whenever our local community changes, so must the ingroup/outgroup label we attach to others. The comparison between multiethnic societies, like India, US and UK, and monoethnic ones, like China, shows that even ethnic foreignness—perhaps the most obvious sort of outgroupness—is just such a provisional tag. In communities that integrate people of different ancestries, the distinction between ethnic ingroups and outgroups no longer matters; other ethnicities may, in fact, appear more interesting. There is hope, then, that racism could be tamed by acquainting our children with people of all shapes and forms, so that everyone in the world looks like family.

## Data Availability

All the data on which the conclusions are based, along with the analysis scripts and annotated outputs, are publicly available via the Open Science Framework and can be accessed at https://osf.io/by2xd. The original data, analyses and materials, as posted by the authors, can be found at https://osf.io/md7nb (US/India Study) and https://osf.io/t476f (UK/China Study).
